# Male infants at risk for ASD have deficit in expressive language at 14 months of age

**DOI:** 10.1192/j.eurpsy.2021.568

**Published:** 2021-08-13

**Authors:** S. Kiselev

**Affiliations:** Clinical Psychology, Ural Federal University, Ekaterinburg, Russian Federation

**Keywords:** autism spectrum disorders, expressive language, Bayley Scales

## Abstract

**Introduction:**

Autism spectrum disorders (ASD) is one of the most common neurodevelopmental disorders. It is known that infants who have older siblings with ASD have a risk for development of this disorder. It is important to study the development of children at risk for ASD to reveal early markers for ASD.

**Objectives:**

The aim of this research was to investigate the neurocognitive abilities in children at risk for ASD at 14 months of age.

**Methods:**

The experimental group included 21 infants at risk for ASD at 14 months (12 boys and 9 girls). The control group included 21 typically developing children. The children from groups were matched for gender and age. The Bayley Scales (3rd Ed.) were used to evaluate the neurocognitive abilities in children.

**Results:**

The results were evaluated by two-way ANOVA, with level of performance in five Bayley scales as dependent variable, with group and gender as between-subjects factors. We did not reveal the significant (p≤0,05) influence of the group and gender on performance in cognitive scale, receptive language, gross and fine motor. However, the infants at risk for ASD performed significantly (p≤0,05) more poorly than infants from control group on expressive language. No differences were found between female infants at risk for ASD and female infants from control group on expressive language.

**Conclusions:**

The obtained results show that male infants at risk for ASD have deficit in expressive language at 14 months of age. It is possible that delay in development of expressive language can be early markers for ASD.

